# Exploring interprofessional collaboration and attitudes of health sciences librarians

**DOI:** 10.5195/jmla.2020.804

**Published:** 2020-07-01

**Authors:** Rachel J. Hinrichs, Caitlin J. Bakker, Tara J. Brigham, Emily C. Ginier, Gregg A. Stevens, Kristine M. Alpi

**Affiliations:** 1 rhinrich@iupui.edu, Assistant Health Sciences Librarian, IUPUI University Library, Indiana University Purdue University Indianapolis (IUPUI), Indianapolis, IN; 2 cjbakker@umn.edu, Research Services Librarian, Health Sciences Libraries, University of Minnesota, Minneapolis, MN; 3 brigham.tara@mayo.edu, Assistant Professor of Medical Education and Medical Librarian, Mayo Clinic Libraries, Mayo Clinic, Jacksonville, FL; 4 eginier@umich.edu, Informationist, Taubman Health Sciences Library, University of Michigan, Ann Arbor, MI; 5 gregg.stevens@stonybrook.edu, Health Sciences Librarian, Health Sciences Library, Stony Brook University, Stony Brook, NY; 6 krisalpi@gmail.com, University Librarian, OHSU Library, Oregon Health & Science University, Portland, OR

## Abstract

**Objective:**

This study assessed health sciences librarians' attitudes toward interprofessional collaboration using the Interdisciplinary Education Perception Scale (IEPS) and gathered information on their involvement with interprofessional activities.

**Methods:**

The authors sent a survey to librarians in the Medical Library Association's (MLA's) Interprofessional Education Special Interest Group and Research Section consisting of the IEPS and questions about their prior and current experiences with interprofessional practice and education (IPE). We compared mean IEPS scores between each MLA group and several other demographic factors to assess differences in attitudes. We also compared librarians' IEPS scores with those of previously published health professional students' IEPS scores and thematically analyzed two open-ended questions.

**Results:**

Health sciences librarians' scores on the IEPS indicated positive attitudes toward IPE. There were no statistically significant differences between any group. Health sciences librarians' mean IEPS score was similar to the mean score of health professions students from a prior study. The most commonly reported interprofessional activity was teaching or facilitating learning activities for health professions students; fewer served on committees or engaged in non-curricular activities such as grand rounds and book clubs.

**Conclusion:**

Health sciences librarians in this study reported positive attitudes toward IPE, in line with the majority of other previously studied health professionals. Years of experience, previous health professional careers, and experience supporting IPE as a librarian had little bearing on the responses to the survey. This suggests that health sciences librarians have positive attitudes toward IPE, regardless of whether they directly support IPE programs or participate in interprofessional activities.

## INTRODUCTION

Team-based, collaborative health care is recognized as an effective model for providing high-quality care, improving communication, and lowering costs [1]. To prepare future health professionals to work in collaborative environments, medical and health sciences schools have begun implementing interprofessional practice and education (IPE) programs. IPE exposes health professions students to each other through structured learning opportunities. The goal is to foster communication skills, an understanding of and respect for other professions, and the ability to problem-solve using a broader, patient-centered perspective [2].

Librarians routinely work with a variety of health professionals in different settings, which creates the opportunity for them to play an important role in IPE and collaborative practice. Librarians' involvement with IPE has primarily been reported at professional conferences [3–6] and in the peer-reviewed literature [7–12]. In 2016, the Medical Library Association (MLA) published the first book about librarians and IPE, including case studies on the development and evaluation of IPE programs and examples from clinical practice [13]. As partners to different health professionals, librarians have roles in IPE in both academic and clinical settings. In academic settings, librarians have focused their efforts on participating in IPE through evidence-based practice (EBP), problem-based learning instruction, non-curricular activities such as book clubs, and provision of a physical space to meet for various disciplines.

Two studies describe how librarians established an interprofessional book club to encourage interprofessional communication, collaboration, and respect between different health professions students and faculty [14, 15]. In a systematic review, Maggio and colleagues suggest using an IPE approach to teach EBP by including students and instructors from different disciplines, including librarians [16]. Some examples included librarian-created, case-based modules to teach evidence-based search skills to sonography and nutrition students [11]; librarians creating online learning modules and using a flipped classroom approach to teach EBP skills to students in eight health professions programs [10, 12]; librarians serving as consultants for medicine and pharmacy students during an integrated EBP course [6]; librarians teaching an interprofessional train-the-trainer course for faculty who are interested in improving their EBP skills [7]; and librarians and library resources being integrated into a problem-based learning IPE module [8]. Librarians' ability to play an important role and forge relationships with many departments on campus is an asset. In some cases, librarians have been asked to serve on IPE committees and to organize campus-wide IPE events [3–5, 17]. Libraries have also served as a space for IPE classes and events [18]. As IPE programs become integrated into the health professions schools and departments on campus, librarians are uniquely positioned to support these initiatives.

In clinical settings, health sciences librarians, including hospital librarians and clinical informationists, have been involved with interprofessional health care teams since the 1970s [19]. The traditional role of clinical librarians is to provide resources and to use their skills as expert searchers to find information relevant to clinical questions [20, 21]. In some cases, librarians expand on this role to include reading, summarizing, and appraising the literature for clinicians [20, 22]. Health sciences librarians also participate in clinical rounding by working with interprofessional health care teams as they discuss cases and move between patient rooms. Librarians' participation in inpatient rounds has been associated with increased clinical questioning and improvements in clinicians' evidence-based medicine (EBM) skills and clinical decision making [19, 23].

Similar to librarians' participation in IPE instruction, health sciences librarians have also been involved with continuing education. Babineau and colleagues were included in an interprofessional continuing education program, in which the librarian retrieved evidence through literature searches, answered clinical questions, and navigated copyright law [9]. In addition to facilitating continuing education programs, Allen and coauthors describe a continuing education symposium attended by librarians as well as nurses that was designed to increase interprofessional collaboration [24]. By participating on health care teams, information professionals have expanded interprofessional collaboration in health care beyond the health professions.

Librarians' exposure to all of the health sciences disciplines provides them with a unique vantage point on the value of working with other health professionals and how to do so effectively. Health sciences librarianship is collaborative by its very nature. Despite librarians' involvement with IPE programs and collaborative practice, no study has assessed librarians' attitudes toward interprofessional collaboration. It is common practice in the IPE literature to assess health professionals' and students' attitudes toward collaboration and the degree to which collaboration takes place using standardized scales [25]. Therefore, the objectives of this pilot study were to:

1. make a baseline assessment of attitudes toward interprofessional collaboration by two known populations of health sciences librarians using a standardized scale,
2. evaluate any differences in attitudes based on experience with IPE and demographic characteristics,
3. compare health sciences librarians' scores to other health professionals' scores on the same scale, and
4. characterize how librarians have taught or supported IPE.

## METHODS

### Study design

This pilot study used a cross-sectional survey design to measure health sciences librarians' attitudes toward interprofessional collaboration and to gather information on interprofessional activities in which librarians were engaged. The North Carolina State University Institutional Review Board (IRB) exempted this study on September 14, 2018, IRB protocol 14309, “Exploring Interprofessional Engagement and Attitudes of Health Sciences Librarians.”

### Population selection and recruitment

To make a baseline assessment of health sciences librarians' attitudes toward interprofessional collaboration, the authors chose 2 populations affiliated with MLA we perceived would be willing to respond to the survey. The primary population studied was the Interprofessional Education Special Interest Group (IPE-SIG) for their likely interest and involvement with IPE. Because the number of librarians in the IPE-SIG was small and we did not know whether the IPE librarians would be substantially different than other types of health sciences librarians, we added the larger Research Section (RS) as a comparison group of health sciences librarians in diverse job roles to test this assumption. As of October 3, 2018, the RS had 252 members, and the IPE-SIG had 104; 20 of these members overlapped. We believed RS members would likely participate in research regardless of their involvement with IPE. We hoped that comparing these 2 populations would allow us to see whether IPE-SIG members' attitudes toward interprofessional collaboration differed from those of health sciences librarians in general.

On October 1, 2018, we sent recruitment emails to the RS and IPE-SIG email lists inviting members to participate in an online, anonymous survey about IPE. The survey was developed in Qualtrics, a secure data-collection program, and no email addresses or otherwise identifying information were requested. The survey was open for one month. We sent reminder emails with the survey link two weeks after the first email and one week before closing the survey.

### Instrument

We selected the Interdisciplinary Education Perception Scale (IEPS) developed by Luecht and colleagues as our standardized measure for perceptions of interprofessional collaboration [26]. We chose the IEPS because several studies found it more appropriate for advanced students with a greater degree of exposure to their own profession, compared to other measures such as the Readiness for Interprofessional Learning Scale (RIPLS) [27, 28]. While our population was professionals, not advanced students, we determined that it was the best fit of the available instruments for measuring interprofessional collaboration. The IEPS has been found to be valid and reliable and has been used with students in many disciplines, including medicine, nursing, occupational therapy, physical therapy, pharmacy, social work, and dietetics [27, 29]. Usually, the IEPS is used to assess attitudes toward interprofessional collaboration before and after an IPE program [8, 30–32], but it has also been used as a standalone survey [29, 33–35].

The IEPS consists of 18 statements and uses a 6-point Likert-type scale ranging from “strongly disagree” to “strongly agree,” with no neutral rating to force an agreement or disagreement with the statement. The summative score indicates the participants' attitude toward interprofessional collaboration, with a higher score being more positive. IEPS has 4 subscales that measure different aspects of interprofessional collaboration: (1) competency and autonomy, (2) perceived need for cooperation, (3) perception of actual cooperation, and (4) understanding of others' value [26]. The maximum score for each subscale is 90, 72, 90, and 72, respectively. The highest score on this scale is 330.

The survey also included a demographics section that gathered information such as years of experience, credentials, current or previous membership in RS and IPE-SIG, and health professions they had worked with as a librarian. Because some health sciences librarians might have unique experiences with IPE due to previous careers, we also asked if they had previously worked as a health professional. Two open-ended questions asked for details on how respondents taught or supported IPE, if at all, and what impact it might have had. The survey instrument is available in the supplemental appendix. Note that we accidentally reversed the direction of the Likert-type scale from the original IEPS and, therefore, reversed the respondents' scores before analyzing the data.

### Survey pretesting

We tested our survey with four library staff members who were not part of the RS or IPE-SIG to assess the time to complete the survey and to identify unclear, redundant, or overlapping questions. The survey took six to eight minutes to complete. We clarified two of the questions and removed a redundant open-ended question.

### Data analysis

The quantitative data were analyzed using R 3.6.0. We identified overlap between our populations (IPE-SIG and non-IPE SIG) using the questions about IPE-SIG and RS membership. If an individual was part of both groups, we assigned them to the IPE-SIG group so that the groups were mutually exclusive. In cases where respondents belonged to neither group, they were classified with the non-IPE-SIG group. Where a response category contained fewer than five respondents, categories were collapsed to protect the privacy of participants. Responses to the IEPS were grouped in subscales and weighted in accordance with Luecht and colleagues [26]. Where respondents did not answer a question, their score for that subscale and overall measure was omitted to avoid skewing results. One-way analysis of variance (ANOVA) was used to assess differences between scores on the IEPS scale and subscales when considering section and SIG membership, previous career as a health professional, years of experience as a librarian, and experience teaching or supporting IPE. We also compared librarians' IEPS score with those of previously published health professions students' scores using ANOVA [29].

Open-ended text responses were independently analyzed by two of the authors (Stevens and Alpi) with Microsoft Excel spreadsheets (Microsoft Excel 2013 [15.0.4981.1000] Microsoft Office [150.0.4981.1000] 32-bit; version 16.18 for Mac), using open coding to identify potential themes [36]. The lists of themes generated by each author were shared, compared, and reduced by consensus to a single set of themes. These were then used by both authors to re-code all of the responses with the final set of themes. Exemplar quotes were selected for each of the themes.

## RESULTS

### Demographics

A total of 65 unique respondents started the survey, 62 of whom responded to at least 1 question. Of the 62 respondents, 38 were exclusively associated with the RS (15.1% response rate), 11 were exclusively associated with the IPE-SIG (10.6% response rate), and 7 belonged to both groups (35.0% response rate). The 7 belonging to both groups were classified as members of IPE for further analysis. Six respondents did not indicate which group they were associated with, if any, and they were classified in the non-IPE-SIG group for further analysis. It is possible that these respondents either skipped the group association questions or are on the email lists for 1 or both of these groups without being members. Demographic information is shown in Table 1.

**Table 1:** Demographics of respondents (n=62)

	n	%
Membership		
Interprofessional practice and education (IPE) group	18	29.0%
Non-IPE group	44	71.0%
Years of experience		
0–5 years	15	24.2%
6–10 years	15	24.2%
11–15 years	16	25.8%
16–25 years	9	14.5%
>25 years	7	11.3%
Previous experience as a nonlibrarian health professional		
Yes	9	14.5%
No	53	85.5%
Previous experience teaching or supporting IPE*		
Yes	27	44.3%
No	24	39.3%
Unsure	10	16.4%
Professions worked with as a librarian†		
Human medicine	47	81.0%
Nursing	45	77.6%
Physical therapy	37	63.8%
Pharmacy	35	60.3%
Public health	32	55.2%
Health administration	29	50.0%
Physician assistant	25	43.1%
Occupational therapy	23	39.7%
Dietetics	18	31.0%
Social work	17	29.3%
Dentistry	15	25.9%
Kinesiology	12	20.7%
Veterinary medicine	7	12.1%
Optometry	6	10.3%

* 61 respondents due to missing data.

† 58 respondents due to missing data.

In addition to the types of health professionals and students given as selections, respondents also reported working with biomedical engineers, chaplains, clinical lab scientists, communication disorders and speech language pathologists, doctors of medical science, emergency medical technicians, genetic counselors, gerontologists, mental health providers, nuclear medicine technicians, radiography technicians, respiratory therapists, sonographists, quality assurance specialists, social scientists, and doctors of several medical specialties: neurology, gastroenterology, psychiatry, and radiology.

### Quantitative results

When considering the impact of years of experience, previous experience as a nonlibrarian health professional, previous experience teaching or supporting IPE, or section and SIG membership on IEPS scores, no statistically significant relationships were found. When considering the potential impact of these factors on subscale scores, only 1 statistically significant relationship was found: librarians reporting previous experience as a nonlibrarian health professional scored lower on the perceived need for cooperation subscale (*F*(1,60)=5.02, *p*=0.03) (Table 2).

**Table 2:** Interdisciplinary Education Perception Scale (IEPS) mean scores and subscale scores

		Total IEPS score (max=330)			1. Competence and autonomy (max=90)			2. Perceived need for cooperation (max=72)			3. Perception of actual cooperation (max=90)			4. Understanding others' value (max=90)		
		Mean score	(SD)	*p*-value	Mean score	(SD)	*p*-value	Mean score	(SD)	*p*-value	Mean score	(SD)	*p*-value	Mean score	(SD)	*p*-value
Membership																
Non-IPE group	44	261.6	(27.0)	0.55	72.7	(10.5)	0.59	62.9	(8.0)	0.93	78.4	(9.0)	0.71	47.4	(8.8)	0.28
IPE group	18	266.0	(23.2)		74.2	(7.9)		62.7	(8.8)		79.3	(6.9)		49.8	(5.4)	
Years of experience																
0–5 years	15	263.7	(27.1)	0.82	74.7	(4.9)	0.80	60.4	(11.7)	0.45	79.1	(5.8)	0.66	50.9	(4.9)	0.37
6–10 years	15	258.6	(34.0)		70.5	(13.3)		63.6	(6.7)		77.0	(12.0)		47.5	(10.9)	
11–15 years	16	261.1	(24.5)		73.3	(9.7)		61.5	(8.3)		77.8	(8.3)		46.4	(8.9)	
16–25 years	9	264.3	(28.6)		73.7	(11.6)		66.0	(4.2)		79.3	(7.1)		45.3	(9.4)	
> 25 years	7	273.0	(12.8)		74.9	(5.8)		65.1	(4.1)		82.7	(5.2)		50.3	(5.1)	
Previous experience as a nonlibrarian health professional																
Yes	9	262.0	(24.6)	0.91	76.4	(7.9)	0.28	57.3	(8.0)	0.03	79.5	(6.4)	0.78	48.0	(8.5)	0.98
No	53	263.1	(26.1)		72.6	(10.0)		63.7	(7.9)		78.6	(8.7)		48.1	(7.9)	
Previous experience teaching or supporting IPE																
Yes	27	266.7	(22.4)	0.16	73.7	(9.1)	0.20	63.8	(7.3)	0.82	80.0	(6.6)	0.54	49.2	(7.3)	0.64
No	24	254.7	(28.8)		71.0	(10.9)		61.8	(9.6)		76.9	(9.1)		46.5	(9.1)	
Unsure	10	272.0	(22.6)		75.3	(6.7)		62.4	(7.6)		79.1	(11.9)		48.4	(7.1)	

In comparison to mean scores gathered by Hawk and colleagues in their administration of the IEPS to different types of health professions students [29], health sciences librarians fell into the mid-range of student scores across the overall scale (263.0±25.7 in a range of 238.9±29.1 to 291.9±18.7). For the subscales, health sciences librarians had a relatively low mean score when considering the perceived need for cooperation and a relatively high mean score when considering perception of actual cooperation (Table 3).

**Table 3:** IEPS mean scores of health sciences librarians compared to health sciences students reported in Hawk and colleagues [29]

Profession	n	Mean score									
		Total IEPS score (max=330)		1. Competence and autonomy (max=90)		2. Perceived need for cooperation (max=72)		3. Perception of actual cooperation (max=90)		4. Understanding others' value (max=72)	
		Score	(SD)	Score	(SD)	Score	(SD)	Score	(SD)	Score	(SD)
Physician assistant	30	291.9	(18.7)	82.8	(6.5)	67.8	(5.5)	82.8	(7.4)	58.5	(6.4)
Osteopathy	141	277.8	(27.4)	80.9	(9.4)	65.4	(9.7)	77.0	(11.2)	54.5	(8.9)
Physical therapy	37	272.0	(21.9)	79.3	(6.5)	66.0	(6.3)	78.4	(7.5)	48.3	(8.0)
Medicine	120	270.9	(24.5)	80.4	(8.8)	66.8	(7.7)	70.9	(10.7)	52.8	(7.9)
Librarians	62	263.0	(25.7)	73.2	(9.7)	62.8	(8.2)	78.7	(8.4)	48.1	(8.0)
Nursing	111	260.6	(28.7)	72.7	(10.2)	64.2	(8.6)	74.2	(9.1)	49.5	(9.4)
Podiatry	37	257.6	(31.7)	72.0	(10.6)	65.8	(7.1)	72.4	(11.9)	47.4	(9.5)
Social work	37	256.8	(19.6)	69.4	(9.0)	65.2	(6.7)	76.1	(6.2)	46.2	(7.0)
Chiropractic	75	238.9	(29.1)	73.4	(9.5)	55.7	(9.1)	66.0	(12.2)	43.9	(10.5)

### Qualitative results

Thirty-three participants responded to one or both of the qualitative questions. Twenty-six unique responses were received for the prompt, “If you answered yes [that you have previous experience teaching or supporting IPE], please briefly state what this IPE learning was and any impact it may have had.” The coding scheme appears in Table 4.

**Table 4:** Code listing for qualitative analysis

Code/Theme	Elements
Activity	Simulation Workshop Evidence-based practice teaching Grand rounds Journal clubs One-shot session Semester-long course Clinical situations Health literacy instruction Book club Purchasing IPE video package Sit on workgroup Small group
Role/Action	Participating Teaching Facilitating Directing course Running Purchasing Designing IPE space
Disciplines	Nursing students Speech language students First-year medicine students First-year pharmacy students Disciplines beyond medicine Residents Second-year undergraduates Students from 4 health sciences schools Research teams
Engagement with other faculty	Co-run with faculty member Clinicians were wonderful
Impact	Varies In progress Minimal/little (2) Positive skill improvement
Institutional focus on IPE	Administration Student selection
Definition of IPE	Not clear Narrow
Preparation for IPE	Faculty development Prior experience
Undervalued/underappreciated	Skills Compensation Perception
Desire for more involvement	Institution-driven Librarian-driven
Respect for librarians	Negative Positive Mixed

One of the IPE roles that librarians reported was teaching and/or facilitating required learning activities for students (61.5%, 16/26). Most health sciences schools covered various disciplines, with nursing students most commonly mentioned across respondents. Several reported a teaching role on librarian-led searching or EBP training offered to an interprofessional audience; for example, “Teaching evidence-based practice principles to residents and nursing students.” Facilitation was commonly part of a nonlibrary course: “For the past 5 years, I have facilitated a semester long required interprofessional education course at my institution. I have also facilitated required interprofessional education days (2 days per year).” A few librarians had moved past facilitation to course direction: “Course director for an Evidence Based Medicine I course taken by first year medicine and pharmacy students. I am also one of two asst. course directors for the EBM II course.”

Almost a third of respondents (30.8%, 8/26) reported membership on an interprofessional-focused committee. Examples came from both academic and hospital settings: “I serve on the steering committee for my institution's first year foundations of interprofessionalism course” and “I sit on a Psycho-Oncology workgroup at my medical center where interprofessional training of social work practices and principles is a key aim.”

Non-curricular activities, such as book clubs and grand rounds participation, were mentioned (26.9%, 7/26). These activities often involved other faculty or clinicians. One respondent in an academic setting stated: “I run an IPE book club with a faculty member.” A hospital-based respondent shared that “I participated in Grand Rounds at the hospital where individuals from a variety of roles throughout the hospital came for a talk on a particular topic or a particular case.”

The request to state the impact of these roles was infrequently addressed by respondents. Only five of the sixteen responses explicitly addressed impact. Comments ranged from having variable or little impact, to being in progress, to having had impact on skills or attitudes; for example, “Impact of these would have to be minimal, but at least the workshop exposed these students to how the team works together.”

Fourteen additional responses were received for the other open question, “Is there anything else you would like to tell us about your interprofessional experiences?” One of the themes that emerged was the desire for more involvement (42.9%, 6/14). This desire was often as simple as saying IPE was expanding at the institution; for example, “I teach in a pharmacy course that we are looking to combine with one for Med students over the next few years so this is a topic of great interest to me.” In other cases, it was an active desire of the librarian: “I have had conversations about the importance of IPE education and IPE related courses, but I have not participated in any courses (formally) or done anything besides conducting literature searches. I hope to change this!” Definitions of interprofessionalism, and the associated question of which professions were invited to participate in interprofessional activities, were raised by a few respondents with quotes such as “Interprofessional understood more narrowly than we would think.”

Institutional focus on IPE enhanced the IPE experience for two respondents in terms of support from administration being key and how that focus permeated the IPE. Four explicitly mentioned engagement with other faculty, and one mentioned the difference in the students: “Students are screened for their interest which helps because they are more open to working with professions like librarians.” In terms of preparation for IPE, the two paths mentioned were participating in faculty education about IPE and prior experience on a health care team: “Previous institution work as part of a health care team gave me practical experience in interprofessional practice.”

Beyond the desire to be more involved, some respondents (35.7%, 5/14) mentioned that they felt undervalued or underappreciated in their IPE programs. Three examples show concerns about staffing, compensation, and perception:

I think my health professors understand the importance of IPE but they aren't actively involving me at this time. They appreciate librarians. But they don't always understand what librarians do anyway. And we librarians are overextended as it is so sometimes I am afraid to offer to do too much more.

I have had generally good experiences but I definitely feel like health librarians are undervalued with our skills. I would like to be more highly compensated for the work that I do in how it helps our organization be more efficient and on the forefront of research.

None of these groups have EVER indicated that a librarian is a member of the IP team. I'm not sure what they think of us beside a support service. Extremely disappointing considering our work to build these relationships.

Finally, the question of what people think of librarians is relevant to the larger theme of respect. Two of the respondents (14.3%) mentioned respect for librarians in IPE. One was positive: “I am honored to be a member of our IPE steering committee in which I helped put together simulation videos involving members of various health professions and assisted with programming for health issues in our community such as the opioid epidemic.” The other was more nuanced, showing that respect seemed to vary by audience: “In my experience, actual researchers often respect librarians, while administrators and new researchers do not.”

## DISCUSSION

This was the first study that we were aware of that investigated health sciences librarians' experiences with interprofessional activities and views of interprofessional collaboration using a standardized measure. In our study, librarians expressed positive attitudes toward interprofessional collaboration, which was similar to the reported outcomes for other health sciences faculty and students [29, 33]. Although it would be more suitable to compare librarians with medical or health sciences faculty, the data from the Giordano and colleagues' study did not allow a direct comparison [33]. Therefore, this analysis focused on comparing the results from our study with one focusing on health sciences students.

The study by Hawk and colleagues provided data for a sample of 588 students in 8 different health professions using the IEPS [29]. In that study, the mean total score on the IEPS was 265.9 out of a maximum 330. Physician assistant students scored the highest (291.9), and chiropractic students scored the lowest (238.9) [29]. The mean total score for librarians in our study was 263.0, which suggested that librarians fell in the middle range of IEPS scores in comparison to tested health professions students. As Hawk and colleagues cautioned, however, it was unclear what statistically significant differences between scores might mean in terms of attitude [29]. That being said, our study suggested that librarians had positive attitudes toward interprofessional collaboration, similar to the average health professions student.

This study also evaluated differences in health sciences librarians' attitudes depending on their experience with IPE and demographic characteristics. By nature of the job, the library profession is very collaborative with other professions already, regardless of whether people have specifically worked in an IPE context. This might explain why we did not find a wide variance in scores when comparing librarians who have and have not previously supported IPE. Furthermore, there were no differences in scores depending on membership in the IPE-SIG, years of experience as a librarian, and previous experience as a nonlibrarian health professional. This implied that regardless of various factors, health sciences librarians overall had positive attitudes toward IPE and were highly collaborative.

In an effort to bring awareness to how health sciences librarians were supporting IPE activities, we also gathered qualitative data. Librarians who were involved with IPE activities at their institutions were most often teaching and facilitating required learning activities for health professions students, being included on an IPE committee, or contributing to non-curricular activities such as grand rounds and book clubs. A small number of participants felt that librarians were undervalued in terms of IPE and wanted more involvement.

Several limitations to the generalizability of these results must be considered. This was a pilot study to test the questionnaire restricted to 2 limited, but known, populations of librarians so that we could calculate response rates. Based on rates of participation in previous surveys of the RS, we estimated that at least 15% of unique librarians across both groups would participate [37]. It was possible that participants who did not opt in to receive emails through the membership discussion lists did not receive the invitation. Furthermore, there was a low response rate from the IPE-SIG, the study population we assumed would have the most positive attitudes toward IPE, which meant we might lack the statistical power to reflect true differences in scores. Another limitation was that the IEPS was originally designed for advanced students with limited experience with their professions, not working professionals. While we do not anticipate that this affected the results, it was possible that an instrument intended for working professionals would give different results. However, we are not aware that such an instrument exists.

In future research, attitudes toward engaging in interprofessional education and practice should be studied from a wider pool of health sciences librarians, including those who are not members of MLA and perhaps not even health sciences librarians, to identify additional perspectives and themes. Additionally, library science students could be surveyed about their interest in collaborating with other professions and whether they are interested in health sciences research. This population would be more in line with previous research on health professions students.

## CONCLUSION

Overall, we found that health sciences librarians had positive attitudes toward interprofessional collaboration, in line with most health professions students who have been studied. Years of experience, previous health professional careers, and experience supporting IPE as a librarian had little bearing on the responses to the survey. Librarians reported involvement with IPE through instruction, non-curricular activities, committee service, and provision of space for collaborative activities. Future research could determine if non–health sciences librarians or library science students have different attitudes toward interprofessional collaboration.

## Data Availability

Data associated with this article are available in the Oregon Health & Science University Library Digital Collections Scholars Archive at 10.6083/ks65hc77z.

## SUPPLEMENTAL FILE

**Appendix:** Librarian interprofessional attitudes and engagement survey instrument
