# Supplementary material for: Exploring interprofessional collaboration and attitudes of health sciences librarians
Source: J Med Libr Assoc. 2020 Jul 1;108(3):440–51. doi: 10.5195/jmla.2020.804 (PMC7441895; doi:10.5195/jmla.2020.804)
Supplement: Supplementary file 1 — Appendix: Librarian interprofessional attitudes and engagement survey instrument [file jmla-108-3-440-s01.pdf]

JMLA Journal of the Medical Library Association

SC

Supplemental content to
J Med Libr Assoc. Jul;108(3):dx.doi.org/10.5195/jmla.2020.804
www.jmla.mlanet.org
© Hinrichs, Bakker, Brigham, Ginier, Stevens, Alpi 2020

Exploring interprofessional collaboration and attitudes of health sciences librarians

Rachel J. Hinrichs, AHIP; Caitlin J. Bakker, AHIP; Tara J. Brigham; Emily C. Ginier; Gregg A. Stevens, AHIP; Kristine M. Alpi, AHIP

APPENDIX

Librarian interprofessional attitudes and engagement survey instrument

Survey introduction:

You are invited to participate in a research study conducted by a team of Medical Library Association (MLA) member researchers who are exploring health sciences librarians' attitudes toward and experiences with interprofessional education and activities (IPEA). We would like everyone to participate regardless of whether you are engaged with IPEA. Your participation will help us better understand how librarians engage in and consider interprofessional education.

Your participation is anonymous and involves responding to thirty items. The survey takes about eight to ten minutes, and if you are interrupted, you can continue in the same browser window later. Each question is optional. Data will be reported in aggregate, possibly at a research symposium or in publication; individual data, with certain details masked, may be made available as required by the journal data sharing policy. There are no risks to participating beyond those that exist in daily life using web browsing software and the possibility of feeling uncomfortable reflecting on your prior engagement or attitude toward IPEA.

By selecting "Yes" and clicking the red arrows on the lower right to enter the survey, you certify that you are eighteen years old or older and are willing to participate.

- o Yes
- o No

How many years have you worked in health sciences librarianship?

- o 0–5 years
- o 6–10 years
- o 11–15 years
- o 16–20 years
- o 21–25 years
- o more than 25 years

Have you worked as a health professional other than as a health sciences librarian?

- o Yes
- o No
- o Not sure

Please list any credentials or degrees you have earned or are in the process of earning in the health professions; enter none if you have not pursued a health-related credential or degree [open response].

JMLA Journal of the Medical Library Association

SC

Supplemental content to
J Med Libr Assoc. Jul;108(3):dx.doi.org/10.5195/jmla.2020.804
www.jmla.mlanet.org
© Hinrichs, Bakker, Brigham, Ginier, Stevens, Alpi 2020

With which of these professions (as students or practitioners) have you worked as a librarian/information professional? Indicate all that apply:

- o Dietetics
- o Dentistry
- o Health administration
- o Kinesiology
- o Medicine, human
- o Medicine, veterinary
- o Nursing (any level through doctor of nursing [DNP] including advanced practice; e.g., nurse practitioner [NP], certified registered nurse anesthetist [CRNA])
- o Occupational therapy
- o Optometry
- o Pharmacy
- o Physician assistant
- o Physical therapy
- o Public health
- o Social work
- o Other: ____

Have you ever participated in IPEA as a librarian or health professional?

- o Yes, as a librarian, but not a health professional
- o Yes, as a health professional, but not as a librarian
- o Yes, as both a health professional and a librarian
- o No
- o Not sure

Are you a member of the MLA Interprofessional Education Special Interest Group?

- o Yes, currently
- o Not currently, but have been previously
- o Never

Are you a member of the MLA Research Section?

- o Yes, currently
- o Not currently, but have been previously
- o Never

Interdisciplinary Education Perception Scale*

Please indicate the degree to which you agree or disagree with the statement by checking the box of the response that best expresses your feeling. Consider “in my profession” to represent health sciences librarianship.

SC
Supplemental content to
J Med Libr Assoc. Jul;108(3):dx.doi.org/10.5195/jmla.2020.804
www.jmla.mlanet.org
© Hinrichs, Bakker, Brigham, Ginier, Stevens, Alpi 2020

The scale is as follows: SA=strongly agree, A=agree, SWA=somewhat agree, SWD=somewhat disagree, D=disagree, SD=strongly disagree.

	SA	A	SWA	SWD	D	SD
1. Individuals in my profession are well-trained						
2. Individuals in my profession are able to work closely with individuals in other professions						
3. Individuals in my profession demonstrate a great deal of autonomy						
4. Individuals in other professions respect the work done by my profession						
5. Individuals in my profession are very positive about their goals and objectives						
6. Individuals in my profession need to cooperate with other professions						
7. Individuals in my profession are very positive about their contributions and accomplishments						
8. Individuals in my profession must depend upon the work of people in other professions						
9. Individuals in other professions think highly of my profession						
10. Individuals in my profession trust each other's professional judgment						
11. Individuals in my profession have a higher status than individuals in other professions						
12. Individuals in my profession make every effort to understand the capabilities and contributions of other professions						
13. Individuals in my profession are extremely competent						
14. Individuals in my profession are willing to share information and resources with other professionals						
15. Individuals in my profession have good relations with people in other professions						
16. Individuals in my profession think highly of other related professions						

JMLA Journal of the Medical Library Association

SC

Supplemental content to
J Med Libr Assoc. Jul;108(3):dx.doi.org/10.5195/jmla.2020.804
www.jmla.mlanet.org
© Hinrichs, Bakker, Brigham, Ginier, Stevens, Alpi 2020

	SA	A	SWA	SWD	D	SD
17. Individuals in my profession work well with each other						
18. Individuals in other professions often seek the advice of people in my profession						

* Adapted from Luecht RM, Madsen MK, Taugher MP, Petterson BJ. Assessing professional perceptions: design and validation of an Interdisciplinary Education Perception Scale. J Allied Health. 1990 Spring;19(2):181–91. Note: The scale presented here is reversed from the original Likert-type scale in Luecht and colleagues, 1990. To follow the original scale, strongly agree should be coded as 6, and strongly disagree as 1.

Have you completed the Interdisciplinary Education Perception Scale (IEPS) or Readiness for Interprofessional Learning Scale (RIPLS) questionnaires before today?

- Yes, both
- Yes, IEPS only
- Yes, RIPLS only
- No
- Not sure

How long ago did you last complete either the IEPS or RIPLS questionnaire?

- 1–3 months
- 4–6 months
- 7–12 months
- 1–2 years
- 2–3 years
- 3+ years
- Not sure when, but I have completed one or both of them
- Never completed either questionnaire

Have you had previous experience teaching or supporting interprofessional education (IPE)?

- Yes
- No
- Not sure

If you answered yes to the previous question, please briefly state what this IPE learning was and any impact it may have had [open response].

Is there anything else you would like to tell us about your interprofessional experiences [open response]?

Thank you for your time in sharing your background and experience with us. If you have questions about your participation, please contact the project coordinator, Kristine M. Alpi, AHIP.
